# Patterns of Sweet Taste Liking: A Pilot Study

**DOI:** 10.3390/nu7095336

**Published:** 2015-08-28

**Authors:** Keiko Asao, Jason Miller, Leann Arcori, Julie C. Lumeng, Theresa Han-Markey, William H. Herman

**Affiliations:** 1 Division of Metabolism, Endocrinology and Diabetes, Department of Internal Medicine, University of Michigan, Domino’s Farms, Lobby G, Suite 1500, 24 Frank Lloyd Wright Drive, Ann Arbor, MI 48106, USA; E-Mails: mill2259@msu.edu (J.M.); leannarcori@gmail.com (L.A.); wherman@med.umich.edu (W.H.H.); 2 Department of Preventive Medicine, University of Tennessee Health Science Center, 66 N. Pauline Street, Suite. 633, Memphis, TN 38163, USA; 3 Department of Pediatrics and Communicable Diseases, University of Michigan, Medical Professional Building, Room D3202, P.O. Box: 5718, 1522 Simpson Road East, Ann Arbor, MI 48109-5718, USA; E-Mail: jlumeng@umich.edu; 4 The Center for Human Growth and Development, University of Michigan, 300 North Ingalls Street, 10th Floor, Ann Arbor, MI 48109, USA; 5 Department of Environmental Health Sciences, University of Michigan, 1415 Washington Heights, Ann Arbor, MI 48109, USA; 6 Michigan Clinical Research Unit, Michigan Institute for Clinical and Health Research, 1500 E. Medical Center Drive, Ann Arbor, MI 48109, USA; E-Mail: hanmark@med.umich.edu; 7 Department of Epidemiology, University of Michigan, 1000 Wall Street, Room 6108/SPC 5714, Ann Arbor, MI 48105-1912, USA; 8 Michigan Center for Diabetes Translational Research, 1000 Wall Street, Room 6108/SPC 5714, Ann Arbor, MI 48105-1912, USA

**Keywords:** taste, sweet taste, liking, sucrose

## Abstract

Two distinct patterns of sweet taste liking have been described: one showing a peak liking response in the mid-range of sucrose concentrations and the other showing a monotonic liking response at progressively higher sucrose concentrations. Classification of these patterns has been somewhat arbitrary. In this report, we analyzed patterns of sweet taste liking in a pilot study with 26 adults including 14 women and 12 men, 32.6 ± 14.5 years of age with body mass index 26.4 ± 5.1 kg/m^2^ (mean ± SD). Sweet taste liking was measured for 10 levels of sucrose solutions (0.035 M to 1.346 M). Participants rated their liking of each solution using a visual analog scale with 0 indicating strongly disliking and 100 strongly liking. The cluster analysis demonstrated two distinct groups: 13 liked relatively low sucrose concentrations and liked high sucrose concentrations less, and 13 liked high sucrose concentrations greatly. If we use the 0.598 M sucrose solution alone and a cutoff liking score of 50, we can distinguish the two clusters with high sensitivity (100%) and specificity (100%). If validated in additional studies, this simple tool may help us to better understand eating behaviors and the impact of sweet taste liking on nutrition-related disorders.

## 1. Introduction

Sweet taste liking may be associated with the types of foods a person consumes [1] but it has not been consistently associated with obesity [2,3,4,5,6]. To delineate the possible association between sweet taste liking and obesity, it is important to carefully characterize inter-individual differences in patterns of sweet taste liking and their associations with food consumption and nutrition-related disorders.

One of the methods to assess sweet taste liking is the forced-choice, paired comparison, tracking procedure [7]. It derives the most preferred concentration in an algorithmic manner. Although previous studies showed that this method is valid [8] and reliable [8,9], the forced-choice, paired comparison, tracking procedure assumes that there is a peak of liking among variable sucrose concentrations. The identified peak, however, would not be valid within a subgroup of people who do not have a clear peak sweetness that they like.

It has been described that there are people who like highly sweet taste and people who do not like highly sweet taste [5,10,11,12,13,14,15,16,17,18]. However, an optimal method, which is simple, reliable, and valid, for distinguishing the two groups has not been established. In the past, an individual’s liking was categorized into patterns visually [16], or using liking at a certain sweetness level, such as sucrose concentration between 0.6 and 0.88 M [12,13,19,20,21,22,23,24,25], although some studies did not explicitly describe the methods for the classification [10,11,26]. Only recently, Kim, *et al.* presented that the patterns of sweet taste liking tested by sucrose and beverages can be classified into three groups using cluster analysis: one group that increases liking while increasing sweetness for sucrose and beverages; the other group that showed the peak liking at the mid-range concentrations for sucrose and beverages; and the last group that showed the former cluster pattern for beverages but the latter cluster pattern for sucrose [18]. Other studies also described more than two patterns, but without using specific quantitative classification methods [16,17]. Several reviews on this topic described these patterns [27,28].

Our pilot study, therefore, had five goals: (1) to evaluate the test-retest reliability over a period of several minutes of liking scores for sucrose across a range of 10 concentrations; (2) to group patterns of sweet taste liking within individuals into meaningful “types”; (3) to identify a liking score threshold and a specific sucrose concentration that can be used to categorize individuals into sweet taste liking pattern “types” with high sensitivity and specificity; (4) to compare the most preferred sucrose concentration and sweet taste liking pattern types assessed by this new rating procedure and the traditional forced-choice, paired comparison, tracking procedure; and (5) to determine if “types” of sweet taste liking are associated with specific demographic and health characteristics.

## 2. Experimental Section

### 2.1. Study Participants

Through a website advertisement, we recruited 30 men and non-pregnant women, 18 years of age or older, who were able to provide informed consent in English. We asked participants to fast overnight and not to brush their teeth, chew gum, or smoke within 1 h of their appointment for their research visit. We scheduled the research visit when the participants had not had, in the previous 36 h, a febrile illness, any type of oral or nasal disease, or a dental procedure. The study protocol was reviewed and approved by the Institutional Review Board (IRB) at the University of Michigan, which served as the IRB of record for the IRB at the University of Tennessee Health Science Center, and all participants provided written informed consent.

This paper presents the data from 26 participants who completed the protocol and had valid data. Three participants were excluded because data were lost due to a software mishap, and one was excluded when he was found to be ineligible after his enrollment. The participants’ characteristics are shown in Table 1. The mean age of the participants was 32.0 (standard deviation (SD) 14.5) years. Nineteen participants (73%) were never-smokers. The mean body-mass index (BMI) was 26.4 (SD 5.1) kg/m^2^. None of the participants had self-reported diabetes mellitus.

**Table 1:** Characteristics of the participants.

	Overall	Cluster		
		Low concentration likers (cluster 1)	High concentration likers (cluster 2)	*p*-value (cluster 1 *vs.* cluster 2)
***N***	26	13	13	
**Age ( years)**	32.0 ± 14.5	29.6 ± 11.9	34.4 ± 16.8	0.41
**Sex**				
Female	14 (54%)	7 (54%)	7 (54%)	1.00
Male	12 (46%)	6 (46%)	6 (46%)	
**Race/Ethnicity**				
Non-Hispanic White	19 (73%)	11 (85%)	8 (62%)	0.38
Others	7 (27%)	2 (15%)	5 (38%)	
**Education**				
Less than bachelor’s degree	13 (50%)	5 (38%)	8 (62%)	0.43
Bachelor’s degree or above	13 (50%)	8 (62%)	5 (38%)	
**Smoking**				
Never	19 (73%)	8 (62%)	11 (85%)	0.47
Former	4 (15%)	3 (23%)	1 (8%)	
Current	3 (12%)	2 (15%)	1 (8%)	
**BMI, kg/m^2^**	26.4 ± 5.1	25.0 ± 4.0	27.8 ± 5.8	0.17
**Duration of fasting (h)**	12.1 ± 4.6	11.7 ± 1.9	12.5 ± 6.3	0.69
**Past medical history**				
No	20 (77%)	10 (77%)	10 (77%)	1.00
Yes	6 (23%)	3 (23%)	3 (23%)	
**Medications**				
None	11 (42%)	5 (38%)	6 (46%)	1.00
Taking any medications	15 (58%)	8 (62%)	7 (54%)	

*p*-values were calculated with Student’s *t*-test for continuous variables and Fisher’s exact test for categorical variables; BMI, body-mass index.

### 2.2. Research Clinic Visit

Each participant attended one 90-min research visit in the morning between July 2012 and September 2012. At the visit, each participant completed a brief questionnaire to provide demographic and health information, including past medical history of heart, lung, kidney, liver diseases, gastrointestinal, oncological, infectious, metabolic (diabetes mellitus), rheumatologic, psychiatric, neurological, dermatological, otolaryngological, and other diseases. We measured each participant’s height and weight using a stadiometer (Easy-Glide Bearing Stadiometer, Perspective Enterprises, Portage, MI, USA) and a calibrated scale (Scale-Tronix Model 6002, White Plains, NY, USA).

### 2.3. Procedure for Measuring Sweet Taste Liking

Each participant underwent three different procedures related to sweet taste liking and perception in the following order: (1) a forced-choice, paired comparison, tracking procedure to determine the most preferred sucrose concentration [8] with modifications [9]; (2) a rating procedure to rate liking of different sucrose solutions on the visual analog scale; and (3) a distinction procedure to assess the participant’s ability to tell the difference between similar sucrose concentrations used during the forced-choice procedure. All procedures occurred during the same study visit. There was a break of 5 min between the forced-choice, paired comparison, tracking procedure and the rating procedure, and a break of 10 min between the rating procedure and the distinction procedure. The results from the distinction procedure are not presented in this paper as the procedure examined only concentrations used during the forced-choice procedure and not the all concentrations used during the rating procedure.

We prepared fresh sucrose solutions using food-grade sucrose (American Sugar Refining Inc., Baltimore, MD, USA) and distilled drinking water (Absopure, Plymouth, MI, USA) at 1.2% to 64.7% weight-to-solvent volume, which are approximately equivalent to the following 10 sucrose concentrations based on the density at 20 °C: 0.035, 0.053, 0.079, 0.118, 0.177, 0.266, 0.399, 0.598, 0.897, and 1.346 M [29]. We chose these concentrations to cover a wide range of sweet tastes and to distinguish inter-individual patterns at a fine resolution based on our pilot study. For all procedures, we served 5 mL of each sucrose solution to participants at room temperature in a disposable plastic cup. Participants tasted each 5 mL [8,9] solution for approximately 5 s, expectorated it, and rinsed their mouths with distilled water after tasting each solution. We provided the solutions at 30- to 60-s intervals. Participants were blinded to the concentration of sucrose they were tasting. Each participant took part in the following two procedures:

(1) Forced-choice procedure: First, we served a pair of sucrose solutions in the middle range of the concentrations (0.118 M and 0.399 M) to each participant. After tasting both solutions, we asked the participant which one he or she liked better. If the participant liked the higher concentration, the next trial was conducted using the selected concentration and the concentration that was two steps higher. If he or she liked the lower concentration, the next trial was conducted using the selected concentration and the concentration that was two steps lower. The trials were continued thereafter with one step intervals, rather than two step intervals. We presented the sucrose solutions in a random order within each pair [9]. The inter-pair intervals were timed for 30 s to 60 s. The series was terminated when the participant chose one concentration as both the higher and lower preferred concentration or if he or she chose the highest or lowest concentration two consecutive times. The maximum number of pairs was set to 12. The series was repeated after a three minute break.

(2) Rating procedure: We served the participant each solution one at a time. Immediately after tasting each solution, participants rated the liking of each sucrose solution on a computerized visual analog scale [30]. The scale had two anchor points at the left and right ends: The left end displayed the phrase “Strongly dislike,” and the right end displayed “Strongly like.” Only the scale for the solution currently being tested was shown on the computer screen. We asked participants to make a mark that represented how much they liked the sucrose solution by clicking a computer mouse. Before beginning the experiment, the participants learned how to use the computer program and the computer mouse during a practice session. The software automatically scored the participants’ responses from 0 (strongly dislike) to 100 (strongly like) [30]. Participants were not aware of the numeric value assigned to their rating on the visual analog scale. After the participants rated their liking for each solution, the screen was changed so that the participants were not able to refer to or modify their ratings for previous solutions. We repeated the entire test procedure after a 3-min break. We served solutions in a different random order generated for each participant by a computer program. The same 10 sucrose solutions were presented, again, but in a newly randomized order. A trained examiner provided supervision and scripted cues to participants throughout the procedure.

### 2.4. Statistical Analysis

We first examined the reproducibility of each individual’s sweet taste liking scores across the two testing series using intraclass correlation coefficients (ICCs). We used mixed models to estimate the ICCs and their confidence intervals [31] using a SAS macro [32].

We next sought to determine whether the patterns of the liking scores of individual participants for the 10 sucrose solutions could be clustered into meaningful groups that represented “types” of sweet taste liking. To account for the variation of scoring scale by subject, we derived a standardized score using the maximum and minimum score recorded for each participant by using the following formula: *(((original score) – (minimum score)) / ((maximum score) – (minimum score))) × 100*. We then drew a response curve for each participant that displayed his/her standardized liking scores for each of the 10 sucrose concentrations. We performed cluster analysis, an unsupervised classification method, with a hierarchical, agglomerative method with Ward’s minimum variance algorithm [33] to classify the liking score curves for all the participants using the individual standardized scores for repeated measurements. We used the cubic clustering criterion (CCC) to determine the most appropriate number of clusters [34]. The number of clusters associated with the highest CCC was considered the best number of clusters [34]. We drew response curves for all participants using standardized scores by cluster to visually confirm the clustering.

We then sought to determine whether a specific cutoff for the liking score for a specific sucrose concentration could be identified as a threshold that would allow sensitive and specific categorization of individual participants into one of the “types” of sweet taste liking identified in the cluster analysis. We calculated the sensitivities and specificities for each of the 10 original (non-standardized) liking scores. We estimated the confidence intervals based on Clopper-Pearson’s exact method [35].

For the comparison between the forced-choice, paired comparison, tracking procedure and the rating on the visual analog scale, we first derived the preferred sucrose concentration based on the two methods for each study subject. For the forced-choice, paired comparison, tracking procedure, the preferred sucrose concentration is defined by the geometric means of the preferred sucrose concentration concluded in the two series. From the rating procedure, the most preferred sucrose concentration is defined by the geometric means of the sucrose concentration of the highest liking score in each of the two series. We estimated the Spearman’s correlation coefficient between the most preferred concentrations measured by the two methods. Furthermore, we assessed the consistency of the classification of high and low concentration likers based on the two methods using kappa-statistics. For this purpose, we classified participants as high concentration likers if their preferred concentration was 0.598 M or higher, and as low concentration likers if their preferred concentration was lower than 0.598 M. We chose the cut-off point 0.598 M from the analysis of sensitivity and specificity to distinguish high and low concentration likers using the rating procedure.

Finally, we examined the participants’ demographic and clinical characteristics by cluster using Student *t*-tests for continuous variables and Fisher’s exact tests for categorical variables. All statistical analyses were performed with SAS 9.2 [35], and the type I error was set at 0.05.

## 3. Results

**Figure 1:**
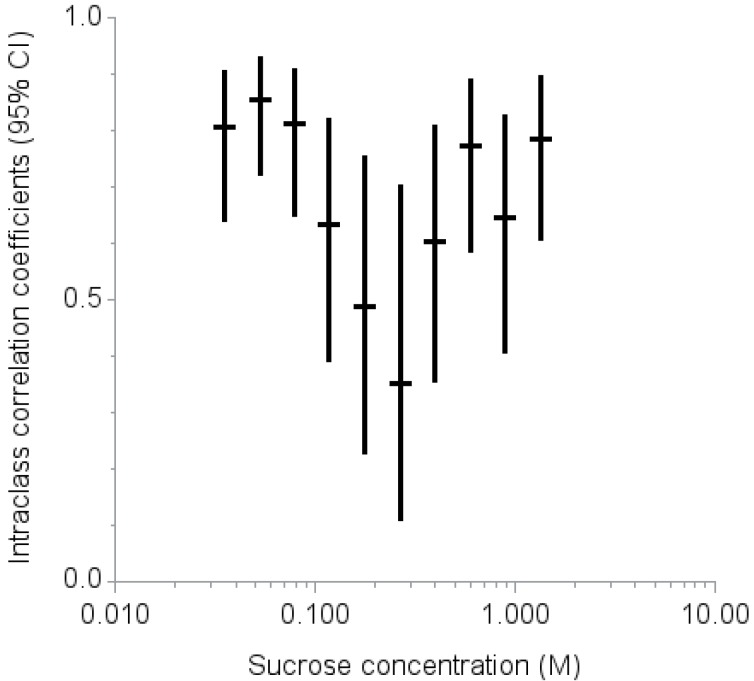
Intraclass correlation coefficients (95% confidence interval) for two repeated measurements of liking scores by sucrose concentration.

The ICCs at each of the 10 levels ranged from 0.35 at 0.266 M to 0.86 at 0.053 M (Figure 1). The intraclass correlation coefficients were higher (ICC 0.70 or above) for low (0.035, 0.053, and 0.079 M) and high (0.598, 0.897, and 1.346 M) sucrose concentrations but lower (ICC less than 0.50) for the middle range of sucrose concentrations (0.177 and 0.266 M).

**Figure 2:**
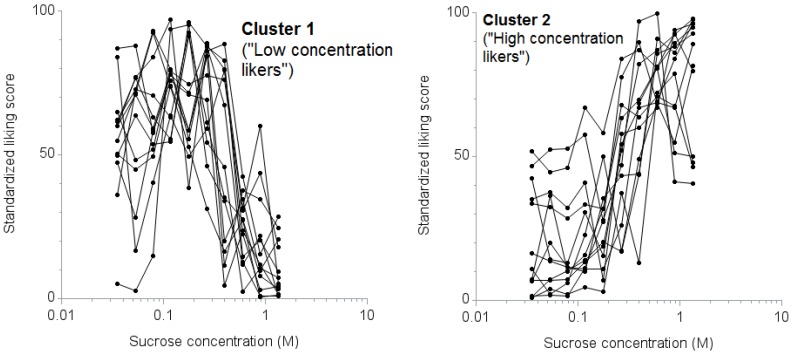
Response curves for liking scores against sucrose concentrations by cluster. The left panel displays the standardized scores of “low concentration likers” (cluster 1), *n* = 13; the right panel displays the standardized scores of “high concentration likers” (cluster 2), *n* = 13.

Cluster analysis showed that the CCC statistic was the highest for two distinct clusters with 13 participants in each (CCC: 0.82). The mean response curves of the individual participants by cluster are shown in Figure 2. We termed the first cluster “low concentration likers” and the second cluster “high concentration likers.”

The liking score of 50 for the 0.598 M sucrose solution best distinguished the “high concentration likers” from “low concentration likers” with high sensitivity (100%, 95% confidence interval (CI): 75% to 100%) and specificity (100%, 95% CI: 75% to 100%) (Table 2).

**Table 2:** Sensitivity and specificity in percentage (95% confidence interval) to identify “high concentration likers” (cluster 2) based on the liking score for one sucrose concentration.

Liking score cutoff	0.399 M Sucrose		0.598 M Sucrose		0.897 M Sucrose		1.346 M Sucrose	
	Sensitivity	Specificity	Sensitivity	Specificity	Sensitivity	Specificity	Sensitivity	Specificity
30	92	23	100	69	100	77	100	92
	(64, 100)	(5, 54)	(75, 100)	(39, 91)	(75, 100)	(46, 95)	(75, 100)	(64, 100)
40	85	46	100	85	100	85	100	92
	(55, 98)	(19, 75)	(75, 100)	(55, 98)	(75, 100)	(55, 98)	(75, 100)	(64, 100)
50	77	62	100	100	85	92	77	100
	(46, 95)	(32, 86)	(75, 100)	(75, 100)	(55, 98)	(64, 100)	(46, 95)	(75, 100)
60	69	62	85	100	69	92	62	100
	(39, 91)	(32, 86)	(55, 98)	(75, 100)	(39, 91)	(64, 100)	(32, 86)	(75, 100)

**Figure 3:**
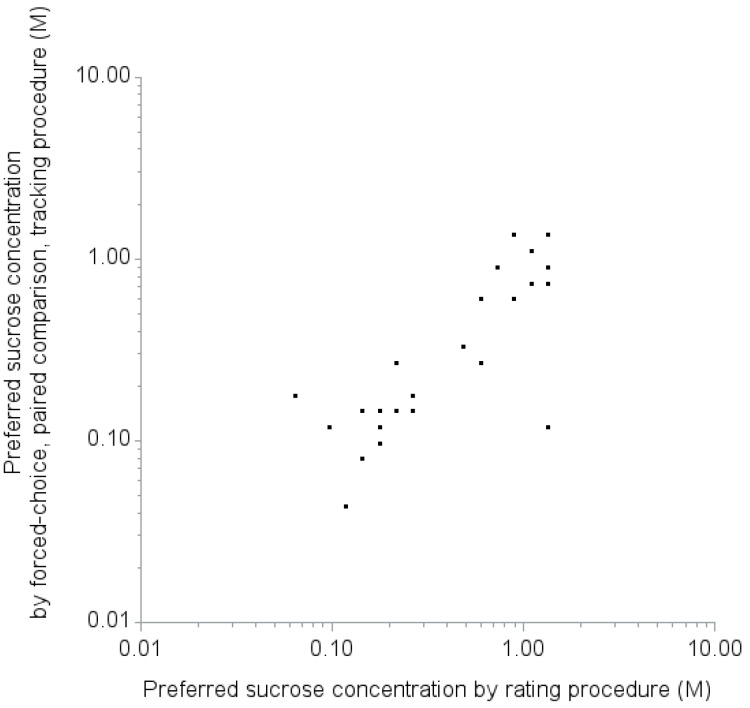
Comparison of the preferred sucrose concentrations measured by rating procedure and forced-choice, paired comparison, tracking procedure. Spearman correlation coefficient was 0.75 (95% CI: 0.51 to 0.88, *p*-value < 0.01).

The preferred sucrose concentrations measured by rating procedure and forced-choice, paired comparison, tracking procedure were consistent (Figure 3). Spearman correlation coefficient was 0.75 (95% CI: 0.51 to 0.88, *p*-value < 0.01). When we classify participants as high concentration likers or low concentration likers with the cut-off for most preferred concentration of 0.598 M measured by the forced-choice, paired comparison, tracking procedure, 16 participants were classified as low concentration likers, while 10 were classified as high concentration likers. Three participants were discordantly classified as low concentration likers by the forced-choice, paired comparison, tracking procedure but high concentration likers by the rating procedure. All 10 participants who were classified as high concentration likers by the forced-choice, paired comparison, tracking procedure were concordantly classified as high concentration likers by the rating procedure. Kappa coefficient was 0.77 (95% CI: 0.53 to 1.00).

Table 1 presents the characteristics of the participants in each cluster. High concentration likers were more likely to be a race/ethnicity other than non-Hispanic White, to be never smokers, to have less education, and to have higher BMI, though none of the differences reached statistical significance.

## 4. Discussion

This study had five main findings. First, based on the ICCs, adults can reliably rate their liking of sucrose at concentrations 0.598 M or above and 0.079 M or below, but are less reliable in the range between 0.177 and 0.266 M. Second, two classifications represent how adults rate sweet taste liking: “high concentration likers” and “low concentration likers.” Third, the classification of the rating of sweet taste liking can be accomplished with high sensitivity and specificity using a cutoff liking score for a sucrose concentration of 0.598 M. Fourth, the classification based on the rating procedure was consistent with findings from a forced-choice, paired comparison, tracking procedure. Lastly, in this small sample, no demographic or health characteristics were significantly associated with being a high concentration liker or low concentration liker.

The patterns of sweet taste liking that we identified are consistent with the report by Thompson, *et al.* [13] and other observers [5,10,11,12,27]. Specifically, there are people who like high sucrose concentrations, and there are people who like relatively low sucrose concentrations and do not like high sucrose concentrations.

This is only the second attempt to classify the pattern of sweet taste liking quantitatively using cluster analysis [18]. In previous studies, the two patterns of sweet taste liking were distinguished using different methods. Some studies used the sucrose concentration 0.6 M among seven sucrose concentrations [12,13] 0.83 M among five sucrose concentrations [19,20,21,24,25], or 0.88 M among three sucrose concentrations [22,23] as the concentration that differentiates the two patterns, but the choices of the cutoff values were not rigorously justified [12,13,19,20,21,22,23,24,25]. Other studies differentiated two patterns ideographically based on the plot of liking scores for five [10], six [11] or 10 [5] sucrose concentrations. Our method provides objective, quantitative evidence to distinguish the patterns of sweet taste liking into two groups. Classifying individuals into one of two patterns is also consistent with the bimodal distribution of sweet taste liking in previous studies [9,13,36]. Furthermore, our analysis offered a specific sucrose concentration to provide a simple method by which to group individuals into one of the two patterns.

A distinct characteristic of high concentration likers in this study was that their sweet taste liking did not diminish at progressively higher concentrations of sucrose. There are a number of possible explanations for the liking of high sucrose concentrations among high concentration likers. First, high concentration likers might have impaired taste perception, including intensity or threshold, and not perceive sweetness as intensely as low concentration likers, although previous studies have shown that the perception of sweetness intensity is not significantly different between the two groups [10,13]. Second, even though we used a wide range of sucrose concentrations, the range may not have been broad enough. The peak of the liking score for high concentration likers may have fallen above the maximum concentrations tested. Future work should include testing liking at even higher sucrose concentrations to determine if a peak is identifiable for high concentration likers. Finally, high concentration likers may have an impaired intrinsic reward system for sweet stimuli. Individuals with personal [20] or family [19,36,37] histories of alcoholism and substance abuse [24] are more likely to be high concentration likers. Liking high sweetness may reflect differences in the endogenous opioid [38] and dopamine [39] systems.

There are three areas of future research in determining the patterns. First, we classified the patterns of the rating of sweet taste liking assuming that the rating reflects sweet taste liking. It is possible that participants had varied interpretations of the word “liking.” For example, some might have interpreted “liking” as a willingness to consume foods with a similar taste. Some participants’ responses may have been biased by beliefs about the healthfulness of foods of varying sweetness. Second, there might be other patterns of sweet taste liking that were not revealed in this study, due in part to the necessarily arbitrary nature of cluster analysis, as well as the small sample size of this study. As a matter of fact, at least two previous literatures listed four patterns, which were named type I through type IV [17,28]: in addition to the two patterns that we found in our study, corresponding to their type II (similar to our “low concentration likers”) and III (similar to our “high concentration likers”), they identified type I as monotonically lower liking with higher sweetness and type IV as the same degree of liking for any sweetness [17,28]. With increased sample size, we may be able to identify these type I and IV patterns and maybe other patterns as well. Third, assessing a possibility of carry-over effect of taste stimuli from the serial tasting will be necessary. Throughout the experiments, we presented the tastants in randomized orders, which would counterbalance carry-over effects in the study participants [40]. Furthermore, based on an *ad hoc* analysis using a mixed model for the original rating score with the sucrose concentration and the indicator of the immediate previous sucrose concentration (higher or lower), there was no sign of significant carry-over effects depending on the previous sucrose concentration (data not shown). Larger future studies concurrently evaluating the conditions relevant for impaired reward systems and sweet liking would provide further insights into this observation.

In this study, a liking score above 50 out of 100 for the 0.598 M sucrose solution best distinguished high concentration likers from low concentration likers. Previous studies used the concentration at 0.83 M to 0.88 M as the most liked sweetness to distinguish the two groups [19,20,21,22,23,36]. Based on our observations, a sucrose concentration around 0.8 M is highly specific for distinguishing high concentration likers from low concentration likers determined by cluster analysis but is not sensitive. Using a liking score for the single sucrose concentration of 0.598 M would simplify and shorten the procedure compared to methods that use multiple solutions to determine the concentration that is best liked. In addition, our study showed relatively high reproducibility of repeated measurements of the liking score at 0.598 M (ICC: 0.77). Future larger studies are necessary to confirm these findings, especially using one concentration test, which eliminates possible carry-over effect of taste stimuli from other concentrations.

We did not identify any significant associations between participants’ characteristics and the patterns of sweet taste liking, similarly to the previous study [18], though our small sample size was likely to have a lower than desired power to detect such effects. Previous studies found: (1) that race/ethnicity other than non-Hispanic White tends to be associated with higher sweet taste liking [41,42]; (2) that there was no association with smoking status [43]; and (3) that obesity was associated with being low concentration likers [5,11]. We did not collect information on lifestyle, including diet or illicit drug use, or on personal and family histories of alcoholism in our study. We were also unable to assess the influence of fasting time or hunger on sweet liking patterns. Relevance of the sweet taste liking patterns to the reward system and actual eating behaviors are yet to be investigated. Larger scale, population-based studies would clarify the distribution of the patterns of sweet taste liking, and aid in investigating other potential clinical correlates of the patterns of sweet taste liking.

## 5. Conclusions

This study is the second attempt to classify patterns of sweet taste liking using cluster analysis. We identified two distinct patterns of sweet taste liking: one in which individuals like ever-higher sucrose concentrations and the other in which individuals like relatively low sucrose concentrations and do not like higher concentrations. A liking score of greater than or less than 50 for a single 0.598 M sucrose solution distinguished between high concentration likers and low concentration likers with high sensitivity and specificity. Knowing the pattern of an individual’s sweet taste liking may help in developing tailored dietary interventions to reduce the caloric consumption from added sugars [44]. Future studies with larger sample sizes to validate our findings and to study the clinical correlates of these patterns are warranted to clarify the meaning of the patterns.
